# Chemical Constituents from Stem Bark and Roots of *Clausena anisata*

**DOI:** 10.3390/molecules171113673

**Published:** 2012-11-20

**Authors:** Jules Lobe Songue, Kouam, Etienne Dongo, Theophile Ngando Mpondo, Robert L. White

**Affiliations:** 1 Department of Chemistry, University of Douala, Cameroon, P.O. Box 24157, Douala, Cameroon; E-Mails: lobejules@yahoo.fr (J.L.S.); jkouam2000@yahoo.fr (K.); t_ngando_mpondo@yahoo.fr (T.N.M.); 2 Department of Chemistry, Dalhousie University, 6274 Coburg Road, P.O. Box 15000, Halifax, NS B3H 4R2, Canada; E-Mail: robert.white@dal.ca; 3 Department of Organic Chemistry, University of Yaounde I, Cameroon, P.O. Box 812, Yaounde, Cameroon; E-Mail: dongoet@yahoo.fr

**Keywords:** Rutaceae, *Clausena anisata*, stem bark and roots, carbazole alkaloids, peptide derivative, phytosterol

## Abstract

Phytochemical investigations on the stem bark and roots of the tropical shrub *Clausena anisata* led to the isolation and characterization three carbazole alkaloids: girinimbine, murrayamine-A and ekeberginine; two peptide derivatives: aurantiamide acetate and *N*-benzoyl-l-phenylalaninyl-*N*-benzoyl-l-phenylalaninate; and a mixture of two phytosterols: sitosterol and stigmasterol. The structures of these compounds were established by nuclear magnetic resonance (^1^H-NMR, ^13^C-NMR, COSY, HSQC, HMQC, HMBC and NOESY) spectroscopy and electrospray ionization mass spectrometry (MS).

## 1. Introduction

*Clausena anisata* (Will). Hook.f. ex .benth., is a tropical shrub or tree growing up to ten meters in height in and on the margins of evergreen forests [1]. Different parts (stem bark, roots, and leaves) of this plant are widely used in traditional medicine to treat many diseases. Traditional healers in Tanzania use *Clausena anisata* against oral candidiasis and fungal infections of the skin [2], whereas in the Temeke district (Daressalam, Tanzania), *Clausena anisata* is used against epilepsy and as an anticonvulsant [3]. In some parts of Africa and in the Philippines, the burning of fresh leaves is utilized to repel mosquitoes [4]. Previous phytochemical investigations on this taxon yielded mostly carbazole alkaloids [5,6,7,8,9,10,11,12], coumarins [13,14,15,16] and limonoids [17]. In continuation of our investigation on the Rutaceae plants [18], we report the identification of seven compounds isolated from *Clausena anisata*.

## 2. Results and Discussion

**Figure 1:**
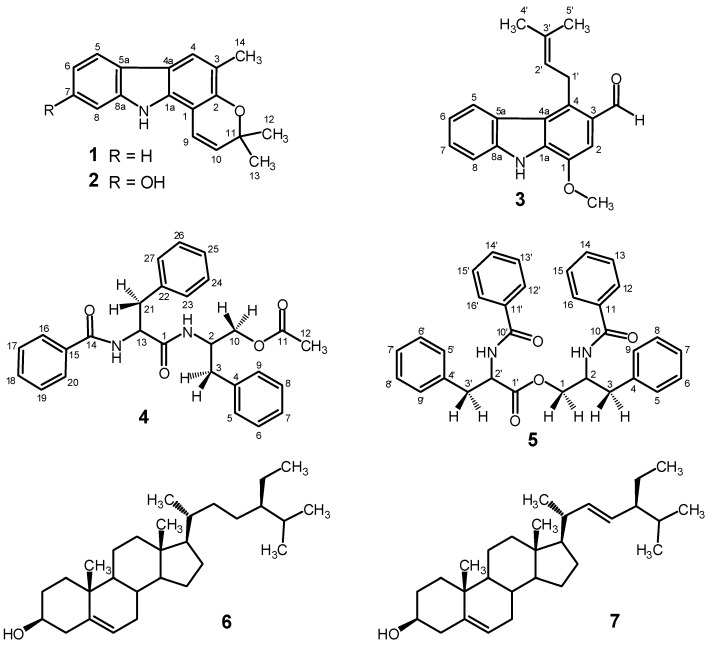
The structures of seven chemical constituents isolated form *Clausena anisata*.

The stem bark and roots of *Clausena anisata* were extracted with methanol. Each extract was subjected to column chromatography and TLC to yield three carbazole alkaloids and two peptide derivatives (Figure 1) identified as: girinimbine (**1**), murrayamine-A (**2**), ekeberginine (**3**), aurantiamide acetate (**4**) and *N*-benzoylphenylalaninyl-*N*-benzoylphenylalaninate (**5**) by spectroscopic methods and direct comparison of spectral data to those published in the literature. A mixture of sitosterol (**6**) and stigmasterol (**7**) was also isolated from both stem bark and roots.

### 2.1. Isolation and NMR (^1^H and ^13^C) Analysis

*Compound* **1** was obtained as a colorless powder from chromatographic fractions 4–7 (5% ethyl acetate in hexane) of stem bark extract. The solid was recrystallized from the eluting solvent to give white crystals (143.5 mg) that had a melting point of 175–176 °C. The purified compound gave a positive reaction with Dragendorff’s reagent. Analysis of the ^1^H-NMR and ^1^H-^1^H-COSY spectra identified a typical ABCD ring system with the benzene ring protons at δ 7.91 (1H, d, *J* = 8 Hz, H-5), 7.38 (1H, d, *J* = 8 Hz, H-8), 7.31 (1H, t, *J* = 7.5 Hz, H-7) and 7.18 (1H, t, *J* = 7.5 Hz, H-6). The broad singlet at δ 7.86 indicated the presence of N-H; the resonances observed at δ 6.63 (1H, d, *J* = 9.5 Hz, H-9), 5.70 (1H, d, *J* = 9.5 Hz, H-10), 1.56 (3H, s, 12-Me) and 1.49 (3H, s, 13-Me) indicated the presence of a chromene ring in the structure. The chromene ring is confirmed by ^13^C-NMR resonances at δ 149.9 (C-2), 129.6 (C-10), 117.4 (C-9), 76.0 (C-11), 27.7 (C-13) and 27.7 (C-12). These data and the distinctive UV spectrum were compared with those of 3,3,5-trimethyl-11*H*-pyrano[3,2-a]carbazole, confirming the structure of **1** as that of the known alkaloid girinimbine, previously isolated from *Murraya koenigii* [19,20] and *Murraya euchrestifolia* [21].

*Compound* **2** was obtained as white crystals from chromatographic fractions 8–11 (10% ethyl acetate in hexane) of stem bark extract. The solid was purified by crystallization from the eluting solvent, yielding a mass of 4.5 mg that melted at 166–167 °C. The purified compound gave positive reactions with Draggendorff’s reagent and ferric chloride, the latter indicating the presence of a phenolic function in the structure. The analysis of ^1^H-NMR and ^1^H-^1^H-COSY spectra identified an ABX mutually coupled proton system at δ 7.75 (1H, d, *J* = 8 Hz, H-5), 6.87 (1H, d, *J* = 2 Hz, H-8) and 6.69 (1H, dd, *J* = 8, 2 Hz, H-6). The broad singlet at δ 7.86 indicated the presence of N-H in the structure. The two three-proton singlets at δ 1.56 (3H, s, 12-Me) and 1.49 (3H, s, 13-Me) together with AB type signals at δ 6.63 and 5.70 (each 1H, d, *J* = 10 Hz, H-9 and H-10) revealed the presence of a 2,2-dimethylpyran ring in the molecule. A downfield shift singlet at δ 7.67 (1H), together with a three-proton singlet at δ 2.33 is due to H-4 and 3-Me of the carbazole skeleton. The presence of a 2,2-dimethylpyran ring was confirmed by ^13^C-NMR chemical shifts at δ 151.2 (C-2), 129.8 (C-10), 117.4 (C-9), 76.0 (C-11), 27.7 (C-13) and 27.7 (C-12) and characteristic UV absorbances. These data, are identical to those in the literature for murrayamine-A, a carbazole alkaloid isolated from *Murraya euchrestifolia* by Wu [22].

*Compound* **3**, isolated as a brown powder (63.2 mg, m.p. 227–228 °C) from fractions 30–33 (15% ethyl acetate in hexane) of stem bark extract, gave a yellow colour with Dragendorff’s reagent. The ^1^H-NMR spectral data of this alkaloid showed signals for an NH (δ 8.65, br s), an aldehyde group (δ 10.46, s), an uncoupled aromatic proton (δ 7.49, s), a methoxyl group (δ 4.05, s), and four aromatic protons of an *ortho*-disubstituted ring at δ 8.15 (dd, *J* = 8.0, 1.3 Hz, H-5), δ 7.52 (dd, *J* = 8.0, 1.3 Hz, H-8), δ 7.46 (td, *J* = 8.0, 1.3 Hz, H-7) and δ 7.30 (td, *J* = 8.0, 1.3 Hz, H-6), typical of a 3-formyl carbazole alkaloid [23]. The UV spectrum of **3** also was a close match to that of 3-formylcarbazole [12]. The arrangement of the aromatic protons in this ABCD ring system was confirmed by the ^1^H-^1^H COSY spectrum, which showed correlations of H-6 with H-5 and H-7, and H-7 with H-8. The ^1^H-NMR spectrum also displayed two olefinic methyl groups (δ 1.91 br s and 1.70 br s), a vinyl proton (δ 5.33, t, *J* = 6.5 Hz) and benzylic methylene protons (δ 4.32, d, *J* = 6.5 Hz), indicating the presence of a prenyl group in the molecule. The ^13^C-NMR and ^13^C-DEPT135 spectra provided evidence that compound **3** possessed an aldehyde (δ_C_ 190.6), a methoxyl group (δ_C_ 55.9), two methyls (δ_C_ 25.8 and 18.5), a benzylic methylene (δ_C_ 27.0), six methines, and eight quaternary carbons. Based on the spectral evidence, the structure of compound **3** was assigned as 1-methoxy-3-formyl-4-(3′-methylbut-2-enyl)carbazole, an alkaloid isolated previously from *Ekebergia senegalensis* by Lontsi *et al*. and named ekeberginine [24].

*Compound* **4** was isolated as white crystals (4.1 mg, m.p. 183–184 °C) from fractions 58–61 of root extract. Analysis of the ^1^H-NMR and ^1^H-^1^H COSY spectra of this compound identified two ABX coupling systems: δ 4.79 (1H, q, *J* = 5.6 Hz, H-13), δ 3.25 (1H, dd, *J* = 13.3, 6.3 Hz, H-21b) and δ 3.08 (1H, dd, *J* = 13.3, 8.4 Hz, H-21a); and δ 4.37 (1H, dddd, *J* = 8.4, 7, 4.9, 4.2 Hz, H-2), δ 3.95 (1H, dd, *J* = 11.9, 4.9 Hz, H-10b) and δ 3.83 (1H, dd, *J* = 11.9, 4.2 Hz, H-10a). A correlation between the proton at δ 4.79 (1H, q, *J* = 5.6 Hz, H-13) and an NH group at δ 6.76 (1H, d, *J* = 7.7 Hz, N-Hb) was observed. Also, the proton at δ 4.37 (1H, dddd, *J* = 8.4, 7, 4.9, 4.2 Hz, H-2) correlated with two other groups of protons at δ 2.77 (2H, m) and δ 5.95 (1H, d, *J* = 8.4 Hz, N-Ha). Analysis of the aromatic proton regions of the ^1^H-NMR and ^1^H-^1^H-COSY spectra revealed the presence of three AA’BB’C coupling systems, each corresponding to a monosubstituted benzene nucleus. The ^13^C-NMR and ^13^C-DEPT135 spectra provided evidence that compound **4** has two amide functions (δ_C_ 171.2 and 168.1), an ester function (δ_C_ 171.8), two benzylic methylenes (δ_C_ 38.4 and 39.4), an oxymethylene (δ_C_ 65.5), a methyl group (δ_C_ 21.8), seventeen methines and three other quaternary carbons. Comparison of these data and the specific rotation with literature values led to the identification of compound **4** as a peptide derivative, aurantiamide acetate, named saropeptate by Ishiguro *et al*. [25]. Aurantiamide acetate (**4**) has been isolated previously from *Aspergillus penicilloides* [26], two alga species (*Cystoseira corniculata* [27] and *Acanthospora specifera* [28]), and several families of higher plants: Euphorbiaceae (*Euphorbia fischeriana* [29] and *Croton hieronymi* [30]), Piperaceae (*Piper aurantiacum* [31,32]), Leguminosae (*Medicago polymorpha* [33] and *Pongamia glabra* [34]), Sterculiaceae (*Pterospermum heyneanum* [35]), Morinagaceae (*Moringa oleifera* [36]), and Rutaceae (*Zanthoxylum setulosum* [37]).

*Compound* **5** was isolated as white crystals (4.6 mg, m.p. 209–210 °C) from fractions 64–68 of root extract. The analysis of spectral data revealed resonances at chemical shifts similar to those of aurantiamide acetate (**4**) suggesting that this compound is also a peptide derivative. The ^1^H-NMR and ^1^H-^1^H COSY spectra displayed three ABX coupling systems at δ 4.65 (1H, dddd, *J* = 8.4, 7, 4.2, 3.5 Hz, H-2), δ 3.03 (1H, dd, *J* = 14, 7 Hz, H-3b) and δ 2.92 (1H, dd, *J* = 14, 8.4 Hz, H-3a); δ 4.94 (1H, q, *J* = 7 Hz, H-2′), δ 3.32 (1H, dd, *J* = 14, 6.3 Hz, H-3′b) and δ 3.24 (1H, dd, *J* = 14, 7 Hz, H-3′a); and δ 4.65 (1H, dddd, *J* = 8.4, 7, 4.2, 3.5 Hz, H-2), δ 4.57 (1H, dd, *J* = 11.9, 3.5 Hz, H-1b) and δ 4.06 (1H, dd, *J* = 11.9, 4.2 Hz, H-1a). Also, a correlation was observed between a proton at δ 4.65 (1H, dddd, *J* = 8.4, 7, 4.2, 3.5 Hz, H-2) and an NH group at δ 6.70 (1H, d, *J* = 8.4 Hz, N-Hb). Similarly, a correlation was observed between a proton at δ 4.94 (1H, q, *J* = 7, H-2′) and an NH group at δ 6.59 (1H, d, *J* = 6.3 Hz, N-Ha). The signals from hydrogens on aromatic rings (δ 7.72–7.23) were compatible with four monosubstituted benzene rings. Thus both compound **5** and aurantiamide acetate (**4**) have *N*-benzoylphenylalanine moieties as the left halves of their structures. Compound **5**, however, was esterified to *N*-benzoylphenylalaninol, as shown by the chemical shifts of H-1, H-2, H-3 and the presence of a second benzamide functionality. The ^13^C-NMR and ^13^C-DEPT135 spectra provided evidence for two amide functions (δ_C_ 168.4 and 168.2), an ester function (δ_C_ 172.9), two benzylic methylenes (δ_C_ 38.5 and 38.3), an oxymethylene (δ_C_ 66.4), twenty-two methines and four quaternary carbons in the structure of compound **5**. These data and the measured specific rotation are consistent with those published for *N*-benzoyl-l-phenylalaninyl-*N*-benzoyl-l-phenylalaninate, also named asperphenamate. Compound **5** has been isolated previously from fungal species: *Aspergillus flavipes* [38], *Anaphalis subumbellata* [39], and the *Penicillum* species *P. canadense* [40], *P. brevicompactum* [41] and *P. megasporum* [42]; and several families of higher plants: Euphorbiaceae (*Croton hieronymi* [30]), Bignognaceae (*Zeyhera digitalis* [43]), Piperaceae (*Piper aurantiacum* [32]), Leguminosaea (*Medicago* *polymorpha* [33] and *Piptadenia gonoacantha* [44]), and Moraceae (*Artocarpus kemando* [45]). 

Dipeptide derivatives are rare and have not been found previously in *Clausena* species. Two other compounds, sitosterol **6** and stigmasterol **7**, were identified by direct comparison of the chemical shifts of their ^1^H and ^13^C-NMR resonances with data published in literature. 

### 2.2. Mass Spectrometry of the Isolated Compounds

Upon electrospray ionization mass spectrometry ( ESI(+)MS ), girinimbine (**1**) was detected as the [M+H]^+^ ion, while all other compounds isolated formed adduct ions with sodium ([M+Na]^+^). Murrayamine-A (**2**), ekeberginine (**3**) and aurantiamide acetate (**4**) also formed [2M+Na]^+^ ions. The prominence of the sodium adducts in the mass spectra contrasts with the low abundance of sodium adducts reported in an extensive survey of the ESI(+)MS of natural products [46] and illustrates the effect of solvent (*i.e.*, methanol *vs.* acetonitrile-formic acid) on ionization behavior. The prominent [M−H]^−^ ions obtained upon ESI(-)MS of girinimbine (**1**), murrayamine-A (**2**), and ekeberginine (**3**) most likely are formed by deprotonation at the carbazole N-H. The gas phase acidity of carbazole (*ca.* 1420 kJ mol^−1^ [47]) is greater than that of aliphatic carboxylic acids (*ca.* 1450 kJ mol^−1^ [47]), which deprotonate readily when subjected to electrospray ionization [48].

The sodium adducts of aurantiamide acetate (**4**) and *N*-benzoylphenylalaninyl-*N*-benzoylphenylalaninate (**5**) yielded distinct product ions upon collision-induced dissociation (CID). For each, rational fragmentation pathways leading to the observed product ions were consistent with the structures assigned.

**Scheme 1:**
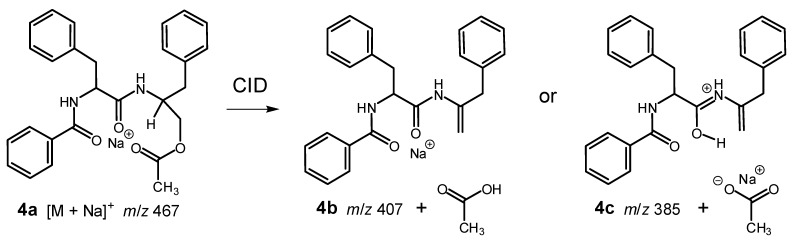
Major MS fragmentation of **4a**, the [M+Na]^+^ ion of aurantiamide acetate (**4**).

CID of the *m*/*z* 467 ion **4a** formed upon ionization of aurantiamide acetate (**4**) yielded five product ions, *m*/*z* 449, 407, 385, 274 and 224. Losses of acetic acid or sodium acetate (Scheme 1) accounted for the major ions at *m*/*z* 407 (**4b**) and 385 (**4c**), respectively. CID of the *m*/*z* 385 ion (generated in-source) yielded the *m*/*z* 224 ion as the major fragmentation product, indicating that the *m*/*z* 224 ion is formed from the *m*/*z* 467 ion via the *m*/*z* 385 ion.

**Scheme 2:**
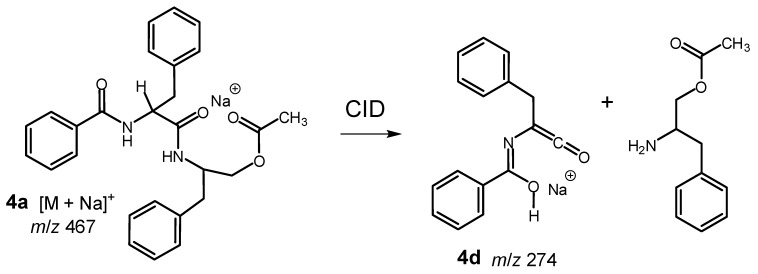
Minor MS fragmentation of **4a**, the [M+Na]^+^ ion of aurantiamide acetate (**4**).

Formation of the *m*/*z* 449 product ion in low abundance indicated loss of water from the *m*/*z* 467 ion **4a** as a minor fragmentation process. A second minor fragmentation process of the *m*/*z* 467 ion **4a** led to formation of the *m*/*z* 274 ion **4d** (Scheme 2). This process is consistent with cleavage of an amide bond and retention of sodium to generate the *m*/*z* 274 product ion from the *N*-benzoylphenylalanine portion of aurantiamide acetate (**4**).

**Scheme 3:**
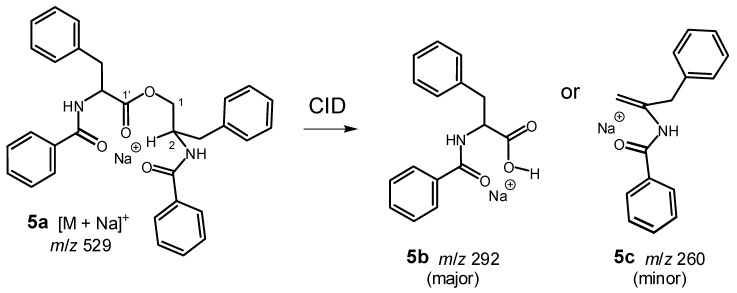
MS fragmentation of **5a**, the [M+Na]^+^ ion of *N*-benzoylphenylalaninyl-*N*-benzoylphenylalaninate (**5**).

Product ions **5b** and **5c** at *m*/*z* 292 (major) and *m*/*z* 260 (minor), respectively, were observed upon CID of the *m*/*z* 529 ion **5a**, formed by ionization of *N*-benzoylphenylalaninyl-*N*-benzoyl-phenylalaninate (**5**) (Scheme 3). The sum of the masses of the two product ions (**5b** and **5c**) equals the mass of the precursor ion **5a** plus that of a sodium ion, indicating that both product ions are sodium ion adducts. Cleavage of the C-1–O bond accompanied by transfer of H-2 to the C-1′ carbonyl oxygen via a six-membered cyclic transition state accounts for the formation of both product ions from the *m*/*z* 529 ion **5a**; the product ion observed depends on which half of ion **5a** retains the sodium ion.

### 2.3. Biosynthesis and Biological Activity

In higher plants, shikimic acid, malonyl-CoA and prenyl phosphate are proposed as the primary biosynthetic precursors of 3-methylcarbazole, an advanced precursor that undergoes oxygenation and prenylation [49] to yield the more highly substituted carbazole alkaloids found in *Clausena* species [5,6,7,8,9,10,11,12] and other plants [49]. With a one-carbon substituent at C-3, the structures of girinimbine (**1**), murrayamine-A (**2**) and ekeberginine (**3**) are consistent with this biogenetic hypothesis.

Previously, several different biological activities have been recognized for compounds **1**–**5**. Cytotoxicity, antitumor activity and induction of apoptosis have been assessed for compounds **1** [6,50,51,52,53], **2** [22], **3** [9] and **5** [54,55], while girinimbine (**1**) also exhibited antimicrobial activity [56], anti-trichomonal activity [57] and cyclooxygenase inhibition [58]. Murrayamine-A (**2**) and aurantiamide acetate (**4**) display antiplatelet aggregation activity [59] and anti-inflammatory properties [36,60], respectively. The enzymes cathepsin [26] and α-glucosidase [61] are inhibited by **4**, whereas **5** is a weak inhibitor of aromatase [62]. Overall, the role of *Clausena anisata* in traditional medicine [2,3] is supported, at least in part, by the biological activities of the alkaloids and peptide derivatives isolated from its stem bark and roots.

## 3. Experimental 

### 3.1. General 

Melting points (uncorrected) were determined on a Gallenkamp melting point apparatus in open capillary tubes. Mass spectra were obtained by electrospray ionization on a Bruker microTOF (accurate mass measurements) and Thermo-Finningan LCQ Duo (tandem mass spectra) mass spectrometers using flow injection in methanol [48]. Collision-induced dissociation (CID) energies are given in parentheses in the arbitrary units (%) supplied by the software. NMR spectra (both 1D and 2D) were acquired on a Bruker AVANCE 500 MHz spectrometer (500.13 MHz for ^1^H and 125.76 MHz for ^13^C) and a Bruker AV-III 700 MHz spectrometer (700.23 MHz for ^1^H and 176.09 MHz for ^13^C) equipped with a 5-mm TCI cryoprobe. Chemical shifts (δ, ppm) are reported relative to TMS as internal standard, and coupling constants (*J*) are given in Hz. Methyl, methylene and methine carbons were distinguished by DEPT experiments. UV spectra were collected in methanol on an Agilent 8345 spectrophotometer, and optical rotations were measured using a Rudolph Instruments Digipol 781 automatic polarimeter. Column chromatography was performed on silica gel (70–230 mesh, 60 Å) using hexane, hexane-ethyl acetate and ethyl acetate as eluents. PTLC was carried out using Merck Si gel 60GF_254_ on glass plates (20 × 20 cm) at a thickness of 0.5 mm. TLC was carried out on Sigma-Aldrich TLC plates, Si gel matrix with fluorescent indicator. Spots on TLC and PTLC plates were visualized under UV light (254 and 366 nm) and by spraying with Dragendorff’s reagent and/or aqueous sulfuric acid (10%).

### 3.2. Plant Material 

The stem bark and roots of *Clausena anisata* were collected from Limbe, south west region, Cameroon, in August 2009. The plant was identified by M. Litonga Ndive Elias, taxonomist at Botanic Garden of Limbe, Cameroon, where the voucher specimen has been deposited.

### 3.3. Extraction and Isolation

Air dried plants (2.4 kg of stem bark and 3.1 kg of roots) were ground to a fine powder and extracted twice with methanol (2 × 10 L, 4 days) at ambient temperature. The stem bark and root extracts were concentrated under reduced pressure to yield dark brown viscous syrups (39 and 35 g, respectively). Each crude extract was subjected to column vacuum chromatography over silica gel and eluted with mixtures of hexane, ethyl acetate and methanol, in order of increasing polarities to give about 110 fractions each. Work up procedures on the fractions afforded three carbazole alkaloids (compounds **1**, **2** and **3**), two peptide derivatives (compounds **4** and **5**), and two phytosterols **6** and **7**, which were identified using spectroscopic methods (1D and 2D-NMR, MS).

*Fractions 4–7* (crude methanol extract of stem bark; eluent: hexane/ethyl acetate/methanol) were combined to give girinimbine (**1**, 143.5 mg) as a colorless powder, C_18_H_17_NO, m.p. 175–176 °C (lit. 175–177 °C [19]). UV λ_max_ nm (log ε): 237 (4.64), 277sh (4.34), 287 (4.57), 327 (3.88), 342 (3.88), 358 (3.82). ^1^H-NMR (500 MHz, CDCl_3_) δ: 7.91 (1H, d, *J* = 8 Hz, H-5), 7.86 (1H, br s, N-H), 7.67 (1H, s, H-4), 7.38 (1H, d, *J* = 8 Hz, H-8), 7.31 (1H, t, *J* = 7.5 Hz, H-7), 7.18 (1H, t, *J* = 7.5 Hz, H-6), 6.63 (1H, d, *J* = 9.5 Hz, H-9), 5.70 (1H, d, *J* = 9.5 Hz, H-10), 2.33 (3H, s, 14-Me), 1.56 (3H, s, 12-Me), 1.49 (3H, s, 13-Me). ^13^C-NMR (126 MHz, CDCl_3_) δ: 149.9 (C-2), 139.9 (C-1a), 134.9 (C-8a), 129.6 (C-10), 124.4 (C-7), 124.0 (C-3), 121.4 (C-4), 119.6 (C-6), 119.4 (C-5), 118.8 (C-5a), 117.4 (C-9), 116.9 (C-4a), 110.5 (C-8), 104.6 (C-1), 76.0 (C-11), 27.7 (C-12), 27.7 (C-13), 16.2 (C-14). ESI(+)MS (relative intensity): *m*/*z* 264 [M+H]^+^; MS/MS (CID 33%) of *m*/*z* 264: *m*/*z* 249 (55), 246 (18), 236 (100), 222 (65); ESI(+)TOF-MS *m*/*z* 264.1394 [M+H]^+^ (264.1383, calculated for C_18_H_18_NO). ESI(-)MS (relative intensity): *m*/*z* 262 [M–H]^−^; MS/MS (CID 38%) of *m*/*z* 262: *m*/*z* 279 (20), 247 (100), 246 (80); ESI(-)TOF-MS *m*/*z* 262.1233 [M–H]^−^ (262.1237, calculated for C_18_H_16_NO).

*Fractions 8–11* (crude methanol extract of stem bark; eluent: hexane/ethyl acetate/methanol) were combined to give murrayamine-A (**2**, 4.5 mg) as white crystals, C_18_H_17_NO_2_, m.p. 166–167 °C (lit. 162–163 °C [22]). UV λ_max_ nm (log ε): 222sh (4.24), 236 (4.35), 278sh (4.12), 287 (4.28), 328 (3.62), 342 (3.64), 357 (3.56). ^1^H-NMR (500 MHz, CDCl_3_) δ: 7.86 (1H, br s, N-H), 7.75 (1H, d, *J* = 8 Hz, H-5), 7.67 (1H, s, H-4), 6.87 (1H, d, *J* = 8 Hz, H-8), 6.69 (1H, dd, *J* = 8, 2 Hz, H-6), 6.63 (1H, d, *J* = 10 Hz, H-9), 5.70 (1H, d, *J* = 10 Hz, H-10), 2.33 (3H, s, 14-Me), 1.56 (3H, s, 12-Me), 1.49 (3H, s, 13-Me). ^13^C-NMR (126 MHz, CDCl_3_) δ: 152.0 (C-7), 151.2 (C-2), 141.1 (C-8a), 139.5 (C-1a), 129.8 (C-10), 124.0 (C-3), 121.1 (C-4), 119.4 (C-5), 118.0 (C-5a), 117.4 (C-9), 115.9 (C-4a), 110.6 (C-6), 106.2 (C-1), 102.5 (C-8), 76.0 (C-11), 27.7 (C-12), 27.7 (C-13), 16.2 (C-14). ESI(+)MS (relative intensity): *m*/*z* 581 (98) [2M+Na]^+^, 302 (100) [M+Na]^+^; ESI(+)TOF-MS *m*/*z* 581.2341 [2M+Na]^+^ (581.2411, calculated for C_36_H_34_N_2_O_4_Na), 302.1120 [M+Na]^+^ (302.1151, calculated for C_18_H_17_NO_2_Na). ESI(-)MS (relative intensity): *m*/*z* 278 [M–H]^−^; MS/MS (CID 36%) of *m*/*z* 278: *m*/*z* 250 (25), 223 (100), 222 (20); ESI(-)TOF-MS *m*/*z* 278.1173 [M–H]^−^ (278.1187, calculated for C_18_H_16_NO_2_).

*Fractions 30–33* (crude methanol extract stem bark; eluent: hexane/ethyl acetate/methanol) were combined to give ekeberginine (**3**, 63.2 mg) as a brown powder, C_19_H_19_NO_2_, m.p. 227–228 °C (lit. 230–231 °C [24]). UV λ_max_ nm (log ε): 240 (4.35), 251sh (4.25), 274 (4.39), 287 (4.27), 343 (3.99). ^1^H-NMR (500 MHz, CDCl_3_) δ: 10.46 (1H, s, 3-CHO), 8.65 (1H, br s, N-H), 8.15 (1H, dd, *J* = 8.0, 1.3 Hz, H-5), 7.52 (1H, dd, *J* = 8, 1.3 Hz, H-8), 7.49 (1H, s, H-2), 7.46 (1H, td, *J* = 8.0, 1.3 Hz, H-7), 7.30 (1H, td, *J* = 8.0, 1.3 Hz, H-6), 5.33 (1H, t, *J* = 6.5 Hz, H-2′), 4.32 (2H, t, *J* = 6.5 Hz, H-1′), 4.05 (3H, s, 1-OMe), 1.91 (3H, s, 5′-Me), 1.70 (3H, s, 4′-Me). ^13^C-NMR (126 MHz, CDCl_3_) δ: 190.6 (3-CHO), 144.2 (C-1), 139.6 (C-8a), 136.7 (C-1a), 134.6 (C-4), 132.9 (C-3′), 126.6 (C-3), 126.1 (C-7), 123.9 (C-4a), 123.2 (C-5), 122.7 (C-5a), 122.4 (C-2′), 120.8 (C-6), 111.5 (C-8), 104.7 (C-2), 55.9 (1-OMe), 27.0 (C-1′), 25.8 (C-4′), 18.5 (C-5′). ESI(+)MS (relative intensity): *m*/*z* 609 (76) [2M+Na]^+^, 316 (100) [M+Na]^+^; MS/MS (CID 24%) of *m*/*z* 316: *m*/*z* 298; ESI(+)TOF-MS *m*/*z* 609.2694 [2M+Na]^+^ (609.2724, calculated for C_38_H_38_N_2_O_4_Na), 316.1293 [M+Na]^+^ (316.1308, calculated for C_19_H_19_NO_2_Na). ESI(-)MS (relative intensity): *m*/*z* 292 (100) [M–H]^−^, 277 (56); MS/MS (CID 30%) of *m*/*z* 292: *m*/*z* 277; MS/MS (CID 30%) of *m*/*z* 277: *m*/*z* 262 (38), 260 (14), 249 (72), 248 (30), 234 (100); ESI(-)TOF-MS *m*/*z* 292.1330 [M–H]^−^ (292.1343, calculated for C_19_H_18_NO_2_).

*Fractions 58–61* (crude methanol extract roots) (eluent: hexane/ethyl acetate/methanol) were combined to give aurantiamide acetate (**4**, 4.1 mg) as white crystals, C_27_H_28_N_2_O_4_, m.p. 183–184 °C (lit. 184 °C [25]). [α${]}_{D}^{21}$ = −34.3° (CHCl_3_, c = 0.14) (lit. −38.8° (CHCl_3_, c = 0.041) [25]) ^1^H-NMR (700 MHz, CDCl_3_) δ: 7.74 (2H, d, *J* = 7.7 Hz, H-16/H-20), 7.55 (1H, t, *J* = 7.7 Hz, H-18), 7.47 (2H, t, *J* = 7.7 Hz, H-17/H-19), 7.31 (2H, d, *J* = 7 Hz, H-23/H-27), 7.28 (2H, d, *J* = 7 Hz, H-24/H-26), 7.25 (1H, t, *J* = 7 Hz, H-25), 7.20 (2H, d, *J* = 7 Hz, H-5/H-9), 7.16 (1H, t, *J* = 7 Hz, H-7), 7.09 (2H, d, *J* = 7 Hz, H-6/H-8), 6.76 (1H, d, *J* = 7.7 Hz, N-Hb), 5.95 (1H, d, *J* = 8.4 Hz, N-Ha), 4.79 (1H, q, *J* = 5.6 Hz, H-13), 4.37 (1H, dddd, *J* = 8.4, 7, 4.9, 4.2 Hz, H-2), 3.95 (1H, dd, *J* = 11.9, 4.9 Hz, H-10b), 3.83 (1H, dd, *J* = 11.9, 4.2 Hz, H-10a), 3.25 (1H, dd, *J* = 13.3, 6.3 Hz, H-21b), 3.08 (1H, dd, *J* = 13.3, 8.4 Hz, H-21a), 2.80 (1H, dd, *J* = 13.3, 8.4 Hz, H-3b), 2.77 (1H, dd, *J* = 13.3, 7 Hz, H-3a), 2.05 (3H, s, H-12). ^13^C-NMR (176 MHz, CDCl_3_) δ: 171.8 (s, C-11, ester), 171.2 (s, C-1, amide), 168.1 (s, C-14, amide), 137.7 (s, C-22), 134.6 (s, C-15), 137.6 (s, C-4), 132.9 (d, C-18), 130.3 (d, C-24/C-26), 130.1 (d, C-23/C-27), 129.8 (d, C-6/C-8), 129.7 (d, C-5/C-9), 129.6 (d, C-17/C-19), 128.2 (d, C-25), 128.0 (d, C-16/C-20), 127.8 (d, C-7), 65.5 (t, C-10), 55.9 (d, C-13), 50.4 (d, C-2), 39.4 (t, C-21), 38.4 (t, C-3), 21.8 (q, C-12). ESI(+)MS (relative intensity): *m*/*z* 911 (12) [2M+Na]^+^, 467 (100) [M+Na]^+^; MS/MS (CID 31%) of *m*/*z* 467: *m*/*z* 449 (10), 407 (100), 385 (62), 274 (8), 224 (4); ESI(+)TOF-MS *m*/*z* 467.1936 [M+Na]^+^ (467.1941, calculated for C_27_H_28_N_2_O_4_Na).

*Fractions 64–68* (crude methanol extract roots; eluent: hexane/ethyl acetate/methanol) were combined to give *N*-benzoyl-l-phenylalaninyl-*N*-benzoyl-l-phenylalaninate (**5**, 4.6 mg) as white crystals, C_32_H_30_N_2_O_4_, m.p. 209–210 °C (lit. 212.5–213 °C [30]). [α${]}_{D}^{22}$ = –73.5° (EtOH, c = 0.11) (lit. –78.7° (EtOH, c = 0.14) [40]). ^1^H-NMR (700 MHz, CDCl_3_) δ: 7.72 (2H, dd, *J* = 7.7, 1.4 Hz, H-12′/H-16′), 7.69 (2H, dd, *J* = 7.7, 1.4 Hz, H-12/H-16), 7.53 (1H, tt, *J* = 7.7, 1.4 Hz, H-14), 7.46 (1H, tt, *J* = 7.7, 1.4 Hz, H-14′), 7.42 (2H, td, *J* = 7.7, 1.4 Hz, H-13/H-15), 7.34 (2H, td, *J* = 7.7, 1.4 Hz, H-13′/H-15′), 7.23–7.28 (10H, aromatic protons on two phenyl rings), 6.70 (1H, d, *J* = 8.4 Hz, N-Hb), 6.59 (1H, d, *J* = 6.3 Hz, N-Ha), 4.94 (1H, q, *J* = 7 Hz, H-2′), 4.65 (1H, dddd, *J* = 8.4, 7, 4.2, 3.5 Hz, H-2), 4.57 (1H, dd, *J* = 11.9, 3.5 Hz, H-1b), 4.06 (1H, dd, *J* = 11.9, 4.2 Hz, H-1a), 3.32 (1H, dd, *J* = 14, 6.3 Hz, H-3′b), 3.24 (1H, dd, *J* = 14, 7 Hz, H-3′a), 3.03 (1H, dd, *J* = 14, 7 Hz, H-3b), 2.92 (1H, dd, *J* = 14, 8.4 Hz, H-3a). ^13^C-NMR (176 MHz, CDCl_3_) δ: 172.9 (s, C-1′; ester C=O), 168.4 and 168.2 (both s, C-10 and C-10′; benzamide C=O), 138.2 (s, C-4), 136.8 (s, C-4′), 135.2 (s, C-11) 134.3 (s, C-11′), 133.0 (d, C-14), 132.4 (d, C-14′), 130.3 (d, C-5/C-9), 130.2 (d, C-5′/C-9′), 129.9 (d, C-13/C-15), 129.7 (d, C-6′/C-8′), 129.7 (d, C-6/C-8), 129.4 (d, C-13′/C-15′), 128.4 (d, C-7′), 128.1 (d, C-12′/C-16′), 128.1 (d, C-12/C-16), 127.8 (d, C-7), 66.4 (t, C-1), 55.5 (d, C-2′), 51.3 (d, C-2), 38.5 (t, C-3′), 38.3 (t, C-3). ESI(+)MS (relative intensity): *m*/*z* 529 [M+Na]^+^; MS/MS (CID 28%) of *m*/*z* 529: *m*/*z* 292 (100), 260 (10); ESI(+)TOF-MS *m*/*z* 529.2102 [M+Na]^+^ (529.2098, calculated for C_32_H_30_N_2_O_4_Na).

## 4. Conclusions

Three carbazole alkaloids [girinimbine (**1**), murrayamine-A (**2**) and ekeberginine (**3**)], two peptide derivatives [aurantiamide acetate **(4**) and *N*-benzoyl-l-phenylalaninyl-*N*-benzoyl-l-phenylalaninate (**5**)] and a mixture of sitosterol (**6**) and stigmasterol (**7**) were isolated from the stem bark and roots of the tropical shrub *Clausena anisata*. Compounds **1**–**5** have known biological activities, and **2**, **4** and **5** have not been found previously as constituents of *Clausena anisata*.
